# Harmonizing CT scanner acquisition variability in an anthropomorphic phantom: A comparative study of image-level and feature-level harmonization using GAN, ComBat, and their combination

**DOI:** 10.1371/journal.pone.0322365

**Published:** 2025-05-09

**Authors:** Shruti Atul Mali, Nastaran Mohammadian Rad, Henry C. Woodruff, Adrien Depeursinge, Vincent Andrearczyk, Philippe Lambin

**Affiliations:** 1 The D-Lab, Department of Precision Medicine, GROW- Research Institute for Oncology and Reproduction, Maastricht University, Maastricht, Netherlands; 2 Department of Radiology and Nuclear Medicine, GROW- Research Institute School for Oncology and Reproduction, Maastricht University Medical Centre+, Maastricht, Netherlands; 3 Institute of Information Systems, University of Applied Sciences and Arts Western Switzerland, Sierre, Switzerland; 4 Department of Nuclear Medicine and Molecular Imaging, Centre Hospitalier Universitaire Vaudois (CHUV), Lausanne, Switzerland; University of Pisa, ITALY

## Abstract

**Purpose:**

Radiomics allows for the quantification of medical images and facilitates precision medicine. Many radiomic features derived from computed tomography (CT) are sensitive to variations across scanners, reconstruction settings, and acquisition protocols. In this phantom study, eight different CT reconstruction parameters were varied to explore image- and feature-level harmonization approaches to improve tissue classification.

**Methods:**

Varying reconstructions of an anthropomorphic radiopaque phantom containing three lesion categories (metastasis, hemangioma, and benign cyst) and normal liver tissue were used for evaluating two harmonization methods and their combination: (i) generative adversarial networks (GANs) at the image level; (ii) ComBat at the feature level, and (iii) a combination of (i) and (ii). A total of 93 texture and intensity features were extracted from each tissue class before and after image-level harmonization and were also harmonized at the feature level. Reproducibility and stability were assessed via the Concordance Correlation Coefficient (CCC) and pairwise comparisons using paired stability tests. The ability of features to discriminate between tissue classes was assessed by measuring the area under the receiver operating characteristic curve. The global reproducibility and discriminative power were assessed by averaging over the entire dataset and across all tissue types.

**Results:**

ComBat improved reproducibility by 31.58% and stability by 5.24%, while GAN increased reproducibility by 8% it reduced stability by 4.33%. Classification analysis revealed that ComBat increased average AUC by 15.19%, whereas GAN decreased AUC by 2.56%.

**Conclusion:**

While GAN qualitatively enhances image harmonization, ComBat provides superior statistical improvements in feature stability and classification performance, highlighting the importance of robust feature-level harmonization in radiomics.

## Introduction

Radiomics is an evolving field in medical imaging that focuses on extracting and analyzing a large number of quantitative features. These features are used to build predictive models for diagnostic, prognostic, and treatment purposes [1,2]. The radiomics hypothesis assumes that complementary knowledge, beyond what can be seen with the naked eye, can be obtained from these extracted quantitative features eventually to aid clinical decision-making. This can be achieved using automated or semi-automated tools [3,4]. By utilizing the information acquired from the extracted radiomic features, it is possible to bridge the gap between radiomics and clinical endpoints [5]. Radiomics has emerged as a result of extensive research in computer-based diagnosis, prognosis, and treatment [6,7]. An extensive amount of data is essential for developing predictive models, which are generally acquired from several hospitals and institutions. Data heterogeneity is a moving target due to continuous upgrades in the scanner and protocol settings. Therefore, it is crucial to have large quantities of data to develop systems that can not only learn disease diversity but also account for the differences between different scanner/protocol settings. Previous research studies [8–13] have highlighted the impact of image acquisition and reconstruction parameters on the reproducibility of radiomic features. Feature variability may also arise from various factors, such as changes in contours or Regions of Interest (ROIs) [14], disparities in inter-observer delineation [14–17], diverse feature extraction algorithms [18], and variations in image processing techniques. Variability on both scanner and protocol settings can potentially compromise not only the reproducibility but also the discriminative power of the radiomic features. Previous investigations [1,13,19,20] have delved into the discriminative capabilities of radiomic features. Nevertheless, it is crucial to acknowledge that the mere reproducibility of a radiomic feature does not inherently assure its discriminative power [12,21]. Thus, it becomes evident that the two facets, namely reproducibility and discriminative power, are intertwined. For instance, a radiomic feature might demonstrate high reproducibility across diverse scanners and protocol configurations while offering little to no discriminative power for the specific problem under consideration [22].

Initiatives have been implemented to standardize computed tomography (CT) image acquisition and reconstruction parameters. For example, the Royal College of Radiologists [23,24] has issued guidelines emphasizing the need for standardizing CT protocols across patient populations, clinical pathways, and cancer imaging, with regular auditing of protocol compliance. Similarly, the European Society of Therapeutic Radiology and Oncology [25,26] panel has issued guidelines for image-guided radiation therapy in prostate cancer. Extending these initiatives to the field of radiomics could help to mitigate the disparities arising from variability across different scanners, protocols, and reconstruction parameters. Radiomics guidelines [5,27–30] typically include standardized protocols for image acquisition, processing, feature extraction, and data analysis to ensure consistency and reproducibility of radiomic studies. These guidelines aim to reduce variability and improve the reliability of radiomic features across different studies and centers. However, due to the extensive variations in protocols across these domains, the practical application of these radiomics guidelines may not be feasible [31]. Certain studies have also exclusively concentrated on identifying reproducible features. For instance, Prayer et al. [32] examined the repeatability and reproducibility of radiomic features extracted from fibrosing interstitial lung disease CT images. Statistical methods such as z-score normalization [33], intensity harmonization methods such as histogram matching [34] and histogram equalization [35] as well as ComBat and its derivatives [36,37] have been previously implemented to rectify batch effects resulting from variations in scanner acquisition protocols and reconstruction parameters. Regarding image-level harmonization, recent studies [38,39] have utilized deep learning approaches. For instance, ImUnity [39] leverages a variational autoencoder Generative Adversarial Network (GAN) for magnetic resonance imaging (MRI) harmonization, significantly improving the quality of harmonized images and classification accuracy across multiple sites. Similarly, a study by Marcadent et al. [38] utilizes a cycle-GAN demonstrating improved reproducibility of radiomic features in chest radiographs, improving diagnostic accuracy for conditions like congestive heart failure.

In this work, we analyze the reproducibility and discriminative power of features extracted from CT images of a phantom scanned under various scanner acquisition parameters, including different reconstruction algorithms, reconstruction kernels, slice thickness, and slice spacing. For instance, the sharpness of the image is impacted by changes made in the convolution criterion [40]. The reconstruction kernel is a crucial parameter within the reconstruction algorithm that defines the sharpness of the images. Furthermore, the variability in slice thickness and slice spacing significantly influences the heterogeneity of the imaging pixel resolution. In this study, we utilize feature-level harmonization with ComBat [41] and image-level harmonization with a GAN. While GANs have been challenged by newer models like diffusion models [42], they remain relevant for image-level harmonization. GANs are capable of generating high-quality images efficiently and learning complex transformations. They are faster in both training and inference compared to diffusion models, which require multiple iterations. Our GAN architecture utilizes a refined shallow Convolutional Neural Network (CNN) as the generator and a critic as the discriminator, incorporating adversarial losses, error losses, and perceptual losses while implementing a Wasserstein GAN [43] with gradient penalty (WGAN-GP) [44]. This configuration aims to stabilize training and enhance the quality of the generated images, addressing common GAN training issues such as mode collapse and unstable dynamics. On the other hand, the ComBat method was originally developed to harmonize gene expression arrays [36], and soon thereafter, it was adapted to harmonize features from medical images [37,45]. We hypothesize that these methods impact the reproducibility and stability, and discriminative power of the radiomic features across various CT scanner protocol settings. Additionally, we introduce a novel ensemble strategy that sequentially combines these two methodologies, channeling the output of the GAN model into the ComBat method. While previous studies have used ComBat for radiomic features harmonization and GANs for image-to-image translation, our study is the first to sequentially integrate these methodologies. As a sub-hypothesis, we propose that this innovative approach leverages the strengths of both methods, potentially improving the harmonization outcomes and ensuring more consistent radiomic analysis.

## Materials and methods

### Data

**Fig 1:**
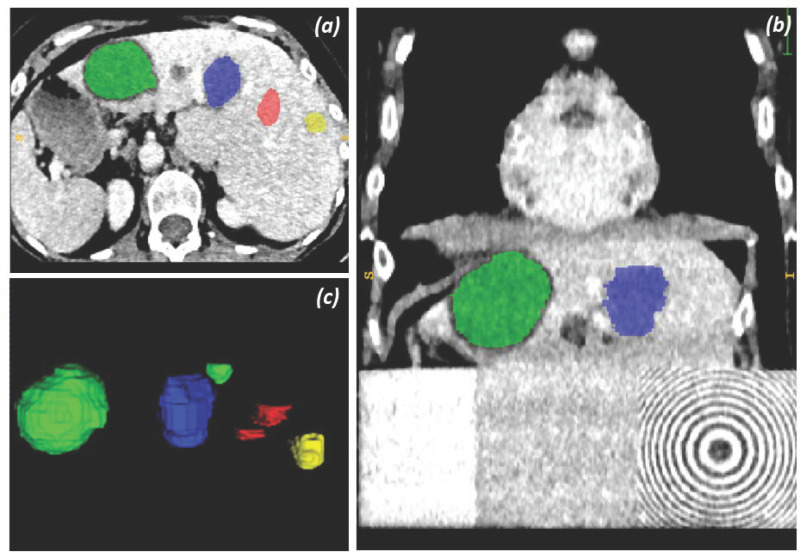
Annotated ROIs in the liver area of a 3D anthropomorphic radiopaque phantom. (a) Presents an axial view of annotated ROIs in the liver region, with green representing benign cysts, blue representing hemangioma, red representing normal liver tissue, and yellow representing liver metastasis from colon carcinoma. (b) Presenting the coronal view of the phantom. (c) Depicting a 3D rendering of the annotated ROIs.

The data used in this study is a three-dimensional anthropomorphic radiopaque phantom [12,46] that was developed to mimic cancer imaging using real patient textural data from the thorax and abdomen, including tumors and lesions. This custom-built phantom was fabricated by printing real patient CT data on paper with an aqueous potassium iodide solution and finally assembling the sheets together. The phantom consists of two sections: an abdominal and a thoracic section. The abdominal section is selected as it is well suited for quantitative analysis with printable densities according to Bach et al. [46,47]. The abdominal section consists of four unique manually annotated ROIs. The annotation process resulted in four semantic ROI binary masks: two normal liver tissues, a metastatic tumor located in the liver originating from a colon carcinoma, a hemangioma, and two benign cysts. Refer to Fig 1 to visualize the phantom with the annotated ROIs in the liver region.

#### Image acquisition and reconstruction.

The Siemens SOMATOM Definition Edge CT scanner (from Siemens Healthineers, Erlangen, Germany) was used to obtain images of the phantom. The acquisition settings of all the CT scans were the same, which included a tube voltage of 120kVp, a helical pitch factor of 1.0, a rotation time of 0.5 seconds, and a tube current time product of 147 mAs without any automatic tube current modulation. This resulted in a volume computed tomography dose index of roughly 10 mGy [12]. This open-source dataset used both filtered back projection (FBP) and iterative reconstruction (IR) algorithms, with IR implemented using ADMIRE (advanced modeled iterative reconstruction) at strength level 3. Additional reconstruction parameters included the kernel (two standard soft tissue kernels for each algorithm), slice thickness in millimeters (1.5, 2, 3), and slice spacing in millimeters (0.75, 1, 2). A total of eight different groups of parameter variations were obtained to evaluate the effect of harmonization using a deep learning technique on the acquired images and a statistical tool (ComBat) on the extracted handcrafted radiomic features. In total, the dataset comprised 240 images, with 30 images per group. Refer to Table 1 for the different reconstruction settings. Out of the eight groups in the dataset, Group 7 was chosen as a reference target group to which the other group images would be harmonized. The motivation for choosing Group 7 as the target group was that IR is a commonly used reconstruction algorithm [48] and the slice thickness and slice spacing are around the average value of the entire dataset.

**Table 1:** Overview of CT reconstruction parameter variations.

Group	Reconstruction Algorithm	Reconstruction kernel	Slice thickness (mm)	Slice spacing (mm)
**1**	FBP	B26f medium smooth ASA	1	0.75
**2**	FBP	B30f medium smooth	1.5	1
**3**	FBP	B30f medium smooth	2	1
**4**	FBP	B30f medium smooth	3	2
**5**	IR	I26f medium smooth ASA	1	0.75
**6**	IR	I30f medium smooth	1.5	1
**7**	IR	I30f medium smooth	2	1
**8**	IR	I30f medium smooth	3	2

#### Pre-processing.

The CT images were first converted from Digital Imaging and Communications in Medicine (DICOM) format to NIfTI format and the masks were converted from RT-struct format to NIfTI format. The ROIs in the masks were re-labeled to have a total of four labels: ROI_1 is normal liver tissue (red in Fig 1), ROI_2 is the benign cysts (green in Fig 1), ROI_3 is hemangioma (blue in Fig 1), and ROI_4 is the colon carcinoma (yellow in Fig 1). The CT images were resampled to isotropic voxels (1.0, 1.0, 1.0) and cropped to remove the CT bed and the empty background of the phantom. The images were further resampled to a standard resolution of (256, 256, 246) to generate preprocessed images of standard resolution to facilitate paired image-to-image training of GANs. The CT images were clipped between [-45, 125] Hounsfield units where the structures within the phantom were qualitatively more distinct for the abdominal organs. This was concluded after viewing the images on the ITK-SNAP platform [49].

### Methods

Two methodological approaches are employed across different domains. Specifically the ComBat [36] method is applied within the feature domain, i.e., on the radiomic features extracted from the CT images. Conversely, GANs are implemented on the images to perform an image-to-image translation from the source domain (Group 1, 2, 3, 4, 5, 6 or 8) to the target domain (Group 7).

#### Feature domain harmonization.

ComBat harmonization is a statistical tool that was originally developed to correct batch effects across gene expression arrays [36]. It is an empirical Bayes-based tool that estimates batch effects while also monitoring the effect of explainable biological variables on the features to be harmonized. To harmonize radiomic features, ComBat calculates a feature value using equation (1) below:


$$y_{ij}=\alpha +\beta .X_{ij}+\gamma_{i}+\delta_{i}.\varepsilon_{ij}$$ (1)

Where:

- $y_{ij}$ represents the value of the radiomic feature for ROI *j* on scanner*i*
- α represents the average value for$y_{ij}$
- β is a vector of regression coefficients that corresponds to each biological covariate, capturing the influence of these variables on the radiomic features
- $X_{ij}$ represents the biological covariates in the form of a design matrix
- $\gamma_{i}$ represents the additive effect scanner *i* on the radiomic features, accounting for systematic differences introduced by different scanners (mean)
- $\delta_{i}$ is the multiplicative scanner effect (variance)
- $\varepsilon_{ij}$ represents the error term that follows a normal distribution with zero mean

The assumption under which ComBat operates is that the mean of site effects ($\gamma_{i}$) follows the same independent normal distributions across all features and the variance of the site effects ($\delta_{i}$) follows independent inverse gamma distributions. $\hat{\alpha }$ and $\hat{\beta }$, the least-squares estimates for each feature are obtained. The empirical Bayes step in ComBat estimates the hyperparameters using the concept of the method of moments, incorporating data from all features [50]. Consequently, the empirical Bayes point estimates denoted as $\gamma_{i}^{*}$ and $\delta_{i}^{*}$, are obtained as the means of posterior distributions. The final ComBat-harmonized data is derived from equation (2) below:


$$y_{ij}^{ComBat}=\frac{Y_{ij}-\hat{\alpha }-\hat{\beta }.X_{ij}-\gamma_{i}^{*}}{\delta_{i}^{*}}+\hat{\alpha }+\hat{\beta }.X_{ij}$$ (2)

ComBat harmonization was implemented on the radiomic features which were derived using the Pyradiomics [51] package. Radiomic features were extracted for all 30 test-retest phantom scans, from each ROI and for each group data and subsequently archived. Shape features were excluded and a total of 93 radiomic features were extracted. Thus, the extraction process yielded a total of 93 radiomic features  ×  8 groups  ×  30 scans  ×  4 ROIs = 89280 features were extracted. ComBat is applied in the feature domain [31] across all batches (or groups) with Group 7 as the reference batch. It is worth noting that ComBat was employed separately for each ROI ensuring effective harmonization of radiomic features within each specific region.

#### Image domain harmonization.

GANs [52] consist of two neural networks: a generator and a discriminator. The generator is responsible for generating synthetic data typically by transforming random noise inputs, while the discriminator aims to distinguish between real data and fake data generated by the generator network. In this particular study, a Pix2Pix GAN model [53] was employed for image-to-image translation, specifically focusing on image harmonization [31] as opposed to utilizing random noise inputs. The Pix2Pix model used in this study is a conditional GAN that is conditioned on input images to generate corresponding output images. Its primary objective is to learn a mapping from the input images to the desired output images. The training process involved pair-wise training, where all batches were trained to a reference batch, i.e., Group 7 images. For example, one GAN training session focused on harmonizing images from Group 1 (G_1) to Group 7 (G_7). This process was repeated with different combinations, resulting in a total of seven GANs trained for pair-wise harmonization: G_x vs G_7, where x in G_x denotes 1, 2, 3, 4, 5, 6, or 8.

**Fig 2:**
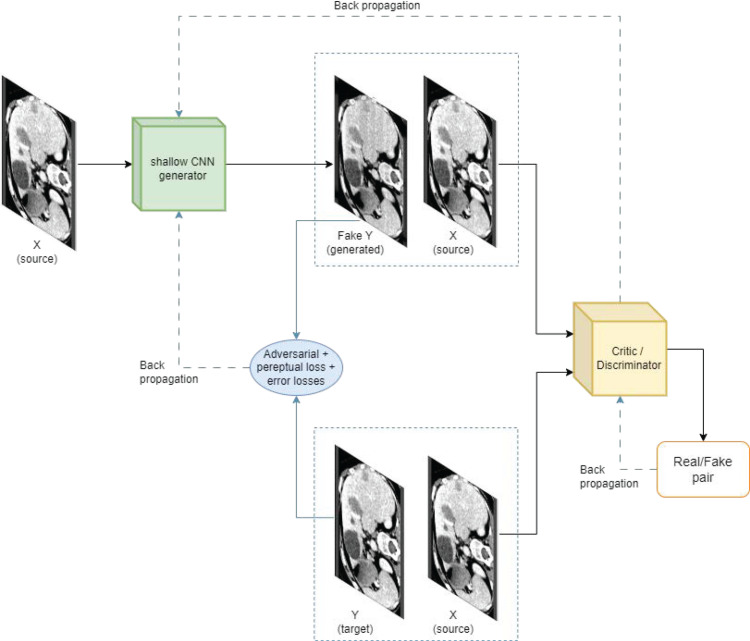
GAN workflow for image level harmonization. The GAN’s generator learns the mapping between the target and source domains, aided by a WGAN-GP-based critic. This process generates harmonized images closely resembling the target domain while preserving source image characteristics.

During training, the generator network was trained on two-dimensional images with a resolution of (256 x 256), aiming to generate output harmonized images of the same size. In addition to the mentioned loss functions, the GAN also incorporated gradient penalty Wasserstein losses [44], to further improve the training stability and encourage convergence between the generator and the discriminator. The discriminator network, originally responsible for classifying images as real or fake, was converted into a critic in the GAN formulation to estimate the Wasserstein distance between the generated and real images. By leveraging these components and techniques, the GAN model in this study aimed to achieve effective image harmonization through pair-wise training, utilizing the Pix2Pix architecture with additional gradient penalty Wasserstein losses (i.e., WGAN-GP) for enhanced training stability and improved convergence. The overall GAN architecture is shown below in Fig 2.

- *Discriminator*

The discriminator comprises four convolutional layers with filter maps numbered [32,64,128,256] each having a kernel size of 5 and stride of 2. Leaky ReLU activation function [54] and Spectral Normalization [55] are applied across these layers to ensure feature identification stability and prevent mode collapse. Dropout layers were applied after each layer to avoid overfitting the discriminator. The last layer of the discriminator aggregates the features into a scalar score, serving as the critic’s output in the WGAN-GP framework.

- Generator

**Fig 3:**
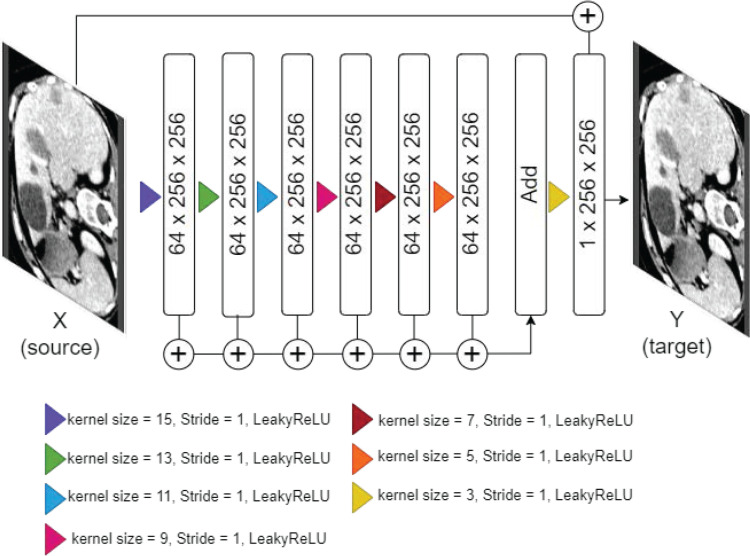
Architecture of the proposed shallow CNN.

The generator model is a shallow CNN specifically designed to capture texture information at different receptive fields (see Fig 3). This is particularly important as changes in scanning acquisition parameters typically result in variations in texture, making this CNN model ideal for our purpose. This shallow CNN consists of seven consecutive convolutional layers with kernel sizes of [15,13,11,9,7,5,3] respectively, a stride of 1, and ReLU activation. The varying kernel sizes allow the network to capture a broad spectrum of texture details. The output of these layers is aggregated and passed to the last convolutional layer using tanh activation. This last layer is further combined with the inputs, followed by ramp activation, to blend the textural features with the original contextual details of the input.

The discriminator’s loss function utilizes the Wasserstein loss with Gradient Penalty (weight=10.0) loss, which includes a gradient penalty term to enforce the Lipschitz constraint and stabilize training. For the generator, the loss function is a combination of several critical components: adversarial, perceptual, textural, and pixel-wise image differences. The perceptual loss is calculated using the VGG19 network [56] from the last convolutional layer in each block. The normalized mean squared error (NMSE) is utilized to measure the pixel-wise image differences between the generated output and the target image, ensuring closer alignment between the two. Peak Signal-to-Noise ratio (PSNR) loss is measured to account for the quality of the reconstructed image compared to the original image. The total generator loss function for the same is as follows:


$$L_{total}=\alpha \times L_{adversarial}+\beta \times L_{perceptual}+\gamma \times L_{L1}+\delta \times L_{nmse}+\varepsilon \times L_{PSNR}$$ (3)

Where α=1.0,β=1000,γ=100,δ=100,ε=100 are the weights for each loss component to enhance the performance of the model. By incorporating these various loss components, the GAN’s loss function aims to capture different aspects of the desired output and guide the training process toward generating visually plausible results. During the training process, we employed an exponential decaying learning rate scheduler utilizing the RMSprop optimizer with an initial learning rate of 0.0002 for the discriminator and the generator with a batch size of 1. The dataset was split into 75–25 for train-test, a decision made to balance effective model training with the need for a robust evaluation given the limited dataset size [57]. To enhance the model’s generalization and robustness, we implemented data augmentation techniques during training. These techniques included random rotations, flipping, and jittering, effectively augmenting the dataset, and mitigating potential overfitting issues [53] due to the limited variety of phantom images within the dataset. The training of the GAN framework was carried out on an NVIDIA GeForce RTX 2080 GPU Ti.

#### Integrated image and feature domain harmonization.

**Fig 4:**
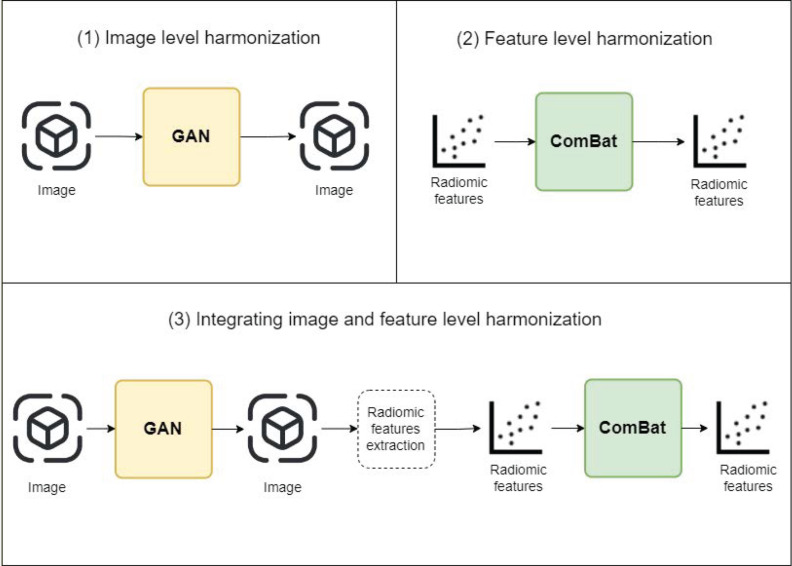
Experimental Setup Overview. This figure presents an overview of the experimental setup conducted in three sub-experiments for harmonizing radiomic data. Sub-experiment 1 involves image-level harmonization using GANs in a pairwise fashion. Sub-experiment 2 focuses on feature-level harmonization through ComBat on radiomic features extracted from ROIs. Sub-experiment 3 combines both image and feature-level harmonization, employing GANs followed by ComBat to achieve comprehensive harmonization.

This study proposes a novel, end-to-end harmonization approach by integrating ComBat, a feature-based harmonization method, with GANs for image harmonization. This strategy addresses the harmonization needed across both image and feature domains. After obtaining the harmonized images from GANs, we proceed to integrate ComBat harmonization. To apply ComBat harmonization, we utilize the harmonized images generated by GANs as input, along with the corresponding ROI masks. The integration of GANs and ComBat harmonization provides a comprehensive approach to address both image harmonization and radiomic feature harmonization challenges. (See Fig 4)

### Experimental setup

The experiment is structured into three distinct sub-experiments, each focusing on different aspects of harmonization. The three experiments are depicted in Fig 4.

#### Image domain harmonization.

In this sub-experiment, image-level harmonization is performed directly on the spatial 2D slices of the phantom data. The harmonization process is achieved using GANs in a pairwise fashion. Specifically, each group is harmonized to a reference group (Group 7) in seven different GAN sessions (each session is Group_x vs Group 7). The GANs aim to generate harmonized images that blend seamlessly with the target domain while preserving the content and characteristics of the source images.

#### Feature domain harmonization.

The second sub-experiment focuses on feature-level harmonization. Radiomic features are extracted from each ROI present in all images for all groups. ComBat, a well-established harmonization method, is applied to adjust the extracted radiomic features using Group 7 as the reference batch. ComBat harmonization accounts for inter-scanner variability and ensures the harmonization of feature distributions across different sources or datasets.

#### Integrated image and feature domain harmonization.

In the third sub-experiment, a holistic approach is adopted by combining both image and feature-level harmonization techniques. First, GANs are applied to harmonize the images, generating harmonized outputs. These harmonized images are then used as inputs to extract radiomic features. Subsequently, the radiomic features extracted from the harmonized images are processed through the ComBat method with Group 7 as the reference batch. This integrated approach aims to achieve enhanced harmonization by addressing both image-level and feature-level variations.

By conducting these three sub-experiments, the study aims to evaluate the effectiveness of each harmonization approach independently and in combination.

### Analysis

#### Radiomic features reproducibility and stability analysis.

To assess the reproducibility and stability of radiomic features before and after harmonization, we utilized three key analysis techniques: Uniform Manifold Approximation and Projection (UMAP) [58] plots, Concordance Correlation Coefficient (CCC) [59] metric and paired comparison tests. UMAP plots visualized the clustering patterns of radiomic features from 8 groups, 30 scans, and 4 ROIs, before and after the harmonization, indicating improved reproducibility if clusters were closer to the reference group (Group 7). CCC metric quantified reproducibility by evaluating the agreement between feature sets, with higher CCC values after harmonization indicating improved consistency. Paired differences were first evaluated for normality using Shapiro-Wilk test: if normality was satisfied paired t-tests were applied, else Wilcoxon signed-rank test was applied. As each feature was compared between reference group (Group 7) and seven other groups, raw p-values were adjusted using Bonferroni correction to account for multiple comparisons. Features with adjusted p-values >0.05 were considered stable. Power analysis (Cohen’s d = 0.5, α = 0.05) confirmed sufficient sample size at the ROI level (achieved power = 1.0), though per-feature analysis indicated lower sensitivity (achieved power = 0.754).

#### Radiomic features discriminative power analysis.

We implemented a comprehensive radiomics pipeline to analyze the discriminative power of the extracted features. Initially, radiomic features were extracted from ROIs 1–4. To enhance the robustness of the feature set, we first removed highly correlated features to reduce redundancy and avoid overfitting. This step is crucial for ensuring that the selected features are both reproducible and relevant. Subsequently, we assessed the discriminative power of the remaining features by conducting classification tasks using a Support Vector Machine (SVM). To optimize the performance of the SVM, we performed a grid search combined with three-fold cross-validation to fine-tune the hyperparameters. The classification performance for each ROI/tissue class was then quantified using the Area under the Receiver Operating Characteristic Curve (AUC).

#### Image quality evaluation.

In evaluating the performance of the GAN, we adopted both qualitative and quantitative approaches. As there is no established consensus in the scientific community on the best evaluation metrics for generative models, we utilized PSNR (Peak Signal-to-Noise Ratio), structural similarity index measure (SSIM), and NMSE (Normalized Mean Squared Error) as metrics to assess the generated image quality comprehensively. For the qualitative analysis, we visually present the input images, the corresponding generated images produced by the GAN, and the target images from the desired domain (i.e., Group 7).

## Results

### Reproducibility and stability analysis

**Fig 5:**
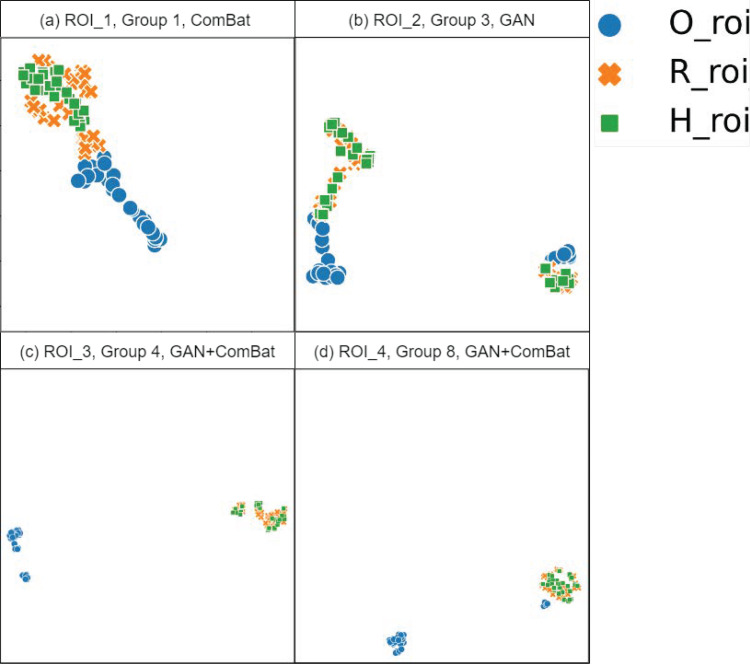
UMAP plots with each subplot visualizing 90 samples (30 scans, for one ROI and one group), described by 93 radiomic features, across harmonization methods.

To visualize the clustering patterns of features, a two-dimensional UMAP reduction was performed in the extracted features. This visualization captures the feature space for the original handcrafted radiomic features (O_roi in blue), harmonized features (H_roi in green), and features from Group 7 (R_roi in orange) as depicted in Fig 5. (a) The proximity of the green and the orange points to each other suggests that ComBat harmonization has effectively shifted the harmonized features towards the reference gestures cluster, demonstrating the impact of the ComBat method. (b) Here, the harmonized features overlap significantly with the reference features despite the proximity to original features and the presence of outliers, reflecting the GAN method. (c) and (d) display a strong overlap of the harmonized features over the reference features cluster, representing the impact of GAN+ComBat harmonization. Please refer to S1–S7 Figs in the S1 File for detailed UMAP plots for each group.

The CCC metric was used against Group 7 features for reproducibility analysis, with a CCC ≥ 0.95 indicating reproducibility. Constant features were excluded from CCC calculations. Table 2 shows that non-harmonized features had an average CCC of 0.92, with 68.57% features exceeding the reproducibility threshold. ComBat harmonization increased the average CCC to 0.99, with 94.29% meeting the threshold. The GAN method resulted in an average CCC of 0.91, with 74.29% reproducible features. The hybrid GAN+ComBat approach matched ComBat, with an average CCC of 0.99 and 94.29% reproducible features.

**Table 2:** CCC calculations averaged across features, ROIs, and groups for each harmonization method.

Method	Average CCC	% of reproducible features
**No harmonization**	0.92	68.57
**ComBat**	0.99	94.29
**GAN**	0.91	74.29
**GAN+ComBat**	0.99	94.29

Table 3 presents the results of stability tests for radiomic features across all four ROIs, comparing the proportion of stable features between non-harmonized and harmonized methods (GAN, ComBat, and GAN+ComBat). ROI_1 shows high stability with no harmonization with 98.92% stable features. In ROI_1, the proportion of stable features increased from 98.92% without harmonization to 100% with both ComBat and GAN+ComBat, while GAN alone resulted in 90.32% stability. For ROI_2, stability improved from 93.55% non-harmonized to 100% with ComBat and GAN+ComBat, whereas GAN alone provided 87.10% stability. In ROI_3, stable features increased from 92.47% non-harmonized to 100% with ComBat and GAN+ComBat, while GAN alone achieved 89.25% stability. Similarly, in ROI_4, stability increased from 94.62% without harmonization to 100% with ComBat and GAN+ComBat, while GAN alone reached 96.77% stability. Overall, ComBat and GAN+ComBat achieved full stability (100%) across all ROIs, while GAN alone showed a slight decline in stability compared to the non-harmonized features.

**Table 3:** ROI-Specific paired stability analysis results.

ROI	No harmonization		GAN		ComBat		Gan+ComBat	
	Stable features	%	Stable features	%	Stable features	%	Stable features	%
**ROI 1**	92/93	98.92	84/93	90.32	93/93	100	93/93	100
**ROI 2**	87/93	93.55	81/93	87.10	93/93	100	93/93	100
**ROI 3**	86/93	92.47	83/93	89.25	93/93	100	93/93	100
**ROI 4**	88/93	94.62	90/93	96.77	93/93	100	93/93	100
**Average**		94.89		90.86		100		100

### Discriminative power analysis

Radiomic features were extracted from the original images to obtain what can be termed as ‘non-harmonized’ radiomic features. These features were then utilized to classify different ROIs through a multi-class classification algorithm employing SVMs, aimed at analyzing their discriminative capabilities. The resulting average scores per ROI show high AUC for ROI_1 (0.88) followed by ROI_4 (0.84), while ROIs 2 and 3 show lower AUC scores of 0.73 (Table 4).S1 Table in S1 File (supplementary material) highlights the discriminative power of the non-harmonized radiomic features, extracted from the original images, across different groups and ROIs through AUC scores. S5 Table in S1 File (supplementary material) highlights pairwise statistical AUC comparisons, where Wilcoxon signed-rank tests are used to assess significant differences between each method.

**Table 4:** ROI-Specific classification scores for all harmonization methods averaged over all groups.

Method	ROI_1	ROI_2	ROI_3	ROI_4	Average AUC
**Non-harmonized**	0.88	0.73	0.73	0.84	0.79
**ComBat**	0.98	0.85	0.85	0.95	0.91
**GAN**	0.79	0.73	0.73	0.83	0.77
**GAN+ComBat**	0.98	0.87	0.88	0.96	0.92

Post-ComBat harmonization, the classification accuracy of the SVM algorithm, as indicated by the AUC scores (refer to Table 4 and S2 Table in S1 File), was substantially enhanced. ComBat increased the average AUC for ROI_1 to 0.98, for ROIs 2 and 3 to 0.85, and for ROI_4 to 0.95, resulting in an overall average AUC of 0.91. The discriminative power of the features extracted from GAN harmonized images has been analyzed in Table 4 and S3 Table in S1 File (refer to supplementary material). For GAN-harmonized images, the average AUC for ROI_1 decreased to 0.79 from 0.88. The average AUC for ROIs 2 and 3 remained at 0.73, while ROI_4 saw a minor drop to 0.83. The GAN+ComBat harmonization approach yielded the highest average AUC of 0.98 for ROI_1. The AUC for ROIs 2 and 3 increased to 0.87 and 0.88, respectively, and ROI_4 reached 0.96. The overall average AUC of 0.92 demonstrates the superior performance of the ensemble approach.

### Image quality analysis

**Fig 6:**
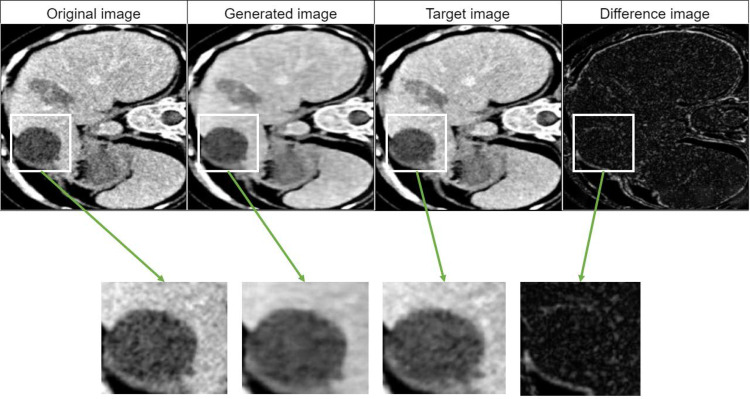
Example of GAN harmonization for Group 2.

Fig 6 shows the results of applying the GAN harmonization method to a single image slice from Group 2. The sequences of images include the original image, before harmonization representing the baseline data; the target image, derived from Group 7, which serves as the reference for harmonization; the generated image, produced by GAN, which aims to replicate the target image’s texture information; the difference image illustrating the disparities between the generated image and target image. The generated image appears to be a less sharp version of the target image indicating a degree of blurring through the GAN method. Refer to S8 Table in S1 File for generated samples of a slice for all groups.

Table 5 gives an overview of the image quality scores following GAN harmonization across the groups with respect to the reference Group 7. The NMSE score ranges from 0.047 in Group 3, indicating the least error, to 0.121 in Group 6, which has the highest error. PSNR scores suggest that Group 3’s images are of the highest quality (32.26 dB), while Group 6’s images are of low quality (23.23 dB) among the groups. The SSIM for most groups is consistent at 0.93, whereas Group 3 again scores the highest (0.97) and Group 5 the lowest (0.89). Overall. Group 3 consistently shows superior image quality post-GAN harmonization, while Group 6 lags, particularly in NMSE and PSNR.

**Table 5:** Image quality scores from GAN harmonization.

Groups	NMSE	PSNR	SSIM
**Group 1**	0.093	25.56	0.93
**Group 2**	0.092	25.49	0.93
**Group 3**	0.047	32.26	0.97
**Group 4**	0.098	24.88	0.92
**Group 5**	0.107	24.08	0.89
**Group 6**	0.121	23.23	0.93
**Group 8**	0.096	25.01	0.93

## Discussion

This study aims to harmonize CT scanner acquisition variability using deep learning and ComBat methodologies presenting a significant advance in the standardization of radiomics data. ComBat [36] was utilized at the feature level to harmonize the radiomic features extracted from these images. For image-level harmonization, GAN [43,53] was utilized to perform an image-to-image translation of paired images to harmonize the images from multiple groups into a reference group. Furthermore, this study investigates a novel ensemble strategy sequentially integrating GAN with the ComBat method. This approach builds upon observations that different harmonization methods can impact radiomic features in complementary ways [39,60]. By sequentially applying WGAN-GP and ComBat, we aimed to integrate their strengths to enhance both the stability and discriminative power of radiomic features. We hypothesized that these image-level and feature-level methods impact the reproducibility and stability, and discriminative power of the radiomic features. We also hypothesized that this integrated approach would improve the harmonization outcomes.

The CCC scores (see Table 2) revealed that the ComBat and GAN+ComBat methods yielded equally high results. Notably, both methods produced similar results in terms of CCC scores, suggesting that the inclusion of ComBat alone may be the driving force behind the enhanced reproducibility seen in the GAN+ComBat approach. This observation shows the potential superiority of ComBat over GANs, despite GANs ability to deliver better concordance (i.e., CCC metric) than non-harmonized features. The stability analysis further demonstrates the robustness of ComBat, with both ComBat and GAN+ComBat showing similar stability enhancements, with a 5.24% increase in the proportion of stable features. In contrast, GAN degraded feature stability compared to the non-harmonized approach (See Table 3) by 4.33% exhibiting limitations, particularly for ROI_1 (normal liver tissue), ROI_2 (benign cysts) and ROI_3 (hemangioma). Interestingly, ROI_1 remained highly stable throughout all conditions, with minimal variability even before harmonization (98.92% stable features). This suggests that ROI_1 may be inherently less sensitive to acquisition differences, requiring less correction compared to other ROIs. However, in the remaining ROIs, stability increased significantly with ComBat and GAN+ComBat, reinforcing the role of feature-level harmonization in mitigating scanner-induced variability. This might suggest that while GAN does not significantly contributes to feature stability, its effectiveness may be strengthened by the statistical power of ComBat harmonization in the GAN+ComBat method.

The comparison of harmonization methods in terms of their influence on the discriminative power of radiomic features reveals distinct outcomes. The non-harmonized features have an overall average AUC of 0.79, setting a baseline for discriminative power. In contrast, the ComBat method significantly improves the discriminative power, achieving an overall average AUC of 0.91, an increase of 15.19% relative to the non-harmonized baseline (p=0.0026; refer S5 Table in S1 File). The GAN method slightly reduces the discriminative power, with an overall average AUC of 0.77 (a 2.53% decrease), and does not differ significantly from the non-harmonized approach (p = 1.0000; S5 Table in S1 File). This suggests that while GAN may have qualitative benefits [ 61–63] (see Fig 6 and S8 Fig in S1 File) in image translation, its capacity to improve the discriminative power of radiomic features is limited when used independently. Conversely, the GAN+ComBat ensemble approach notably improves the discriminative power, reflected by the highest overall average AUC of 0.92 (See Table 4 and S4 Table in S1 File). While the GAN+ComBat ensemble approach improves the discriminative capacity of features by 16.46%, and significantly outperforms the non-harmonized approach (p = 0.0016). Further pairwise comparisons show that ComBat significantly outperforms GAN (p = 0.0098), and GAN+ComBat significantly outperforms GAN (p = 0.0043). However, ComBat does not significantly differ from GAN+ComBat (p = 0.1031). Overall, these results confirm that ComBat-based strategies (either ComBat alone or GAN+ComBat) provide significant improvements in discriminative power over non-harmonized data, with GAN+ComBat achieving the highest overall AUC. These observations present a new hypothesis for future research, which could potentially demonstrate significance with larger datasets and further refinement of such model integration techniques. While our study addresses the harmonization of radiomic features using phantom data, it primarily focuses on distinguishing between healthy and non-healthy tissues. The absence of pathological information in the phantom dataset limited our ability to perform more complex clinical correlations. Power analysis confirmed sufficient sample size at the ROI level (achieved power = 1.0), but feature-level comparisons indicated lower power (0.754), suggesting that additional data may enhance statistical robustness. Future work utilizing patient data with detailed pathological information could further validate and extend the clinical applicability of our harmonization approach.

Interestingly Group 3 exhibited exceptional results, in the aspect of image quality from GAN harmonization (refer to Fig 6 and Table 5). This is potentially due to the factor that the slice thickness and spacing parameters are well-matched with the reference group. The correlation suggests that such acquisition parameters could influence the reproducibility and stability of the extracted radiomic features [12]. This observation leads us to a new hypothesis: that these acquisition parameters critically impact image harmonization outcomes and targeting one type of variability at a time may be more effective than simultaneously addressing multiple scanner variabilities. It is noteworthy to mention the training duration required for GANs, which, in our study, involved multiple training iterations for each group. The extensive time investment for training GANs is a practical consideration for future applications, especially in clinical settings where rapid image processing is often required. It is also important to consider other resource demands such as the need for high-performance GPUs, their cost, and CO_2_ footprint. GANs are also “notoriously unstable and sensitive” during training, which often requires careful hyperparameter tuning and experimentation [64,65]. Additionally, GANs also suffer from mode collapse issues [66] and convergence failure [64].

Several studies [67–72] have utilized ComBat to harmonize radiomic features. More recently, Lee et al. [73] showed that the stability measurement of radiomic features could serve as an evaluation metric in training a GAN to denoise CT images. The image was randomly divided into ten patches, from which radiomic features were extracted, including first-order features, texture features, and wavelet features. To assess the reproducibility of radiomics, the CCC was calculated between the source and target image patches, using 0.85 as a threshold; features exceeding this threshold were considered reproducible. This approach not only confirmed the feature stability but also enabled the fine-tuning of the GAN’s hyperparameters. Our study explores two methods from two different domains; one is a method (ComBat) applied to the features, while the other (GAN) is applied directly to the images. Feature-level harmonization, such as ComBat, effectively removes batch effects and biases, ensuring stable and reproducible radiomic features. However, it might exclude some subtle informative features and depend heavily on annotated data. Image-level harmonization using deep learning approaches like GANs can better preserve spatial and textural information and improve feature consistency across data heterogeneity but is computationally intensive and may introduce unnatural artifacts if not properly implemented. Moving forward, we plan to integrate radiomics reproducibility assessments into GAN training to prioritize feature harmonization.

Previous studies have demonstrated the effectiveness of ComBat in harmonizing radiomic features across different datasets and have explored the use of GANs for image translation. Our study advances the field by uniquely combining these two methodologies in a sequential approach. Whereas prior research typically focused on either feature-level or image-level harmonization independently, our work integrates both levels to evaluate their complementary effects on the stability and discriminative power of radiomic features. However, our study’s reliance on phantom data rather than real patient scans may limit the direct clinical applicability of our findings. While phantoms are valuable for standardized testing, they cannot fully capture the complexity of human pathology. Future work should, therefore, focus on applying these harmonization techniques to patient datasets to confirm their effectiveness in a clinical setting. While this study investigated models trained on paired data, it would also be worthwhile to develop generalizable models on unpaired data.

Additionally, the influence of other CT scanning parameters, such as dose, tube voltage, and pitch, should also be considered, as they could also impact the characteristics of radiomic features [12]. This study used data acquired from a single CT scanner, which allowed for controlled analysis of harmonization methods but does not encompass the full range of imaging conditions encountered across different scanners. As iterative reconstruction algorithms vary considerably between manufacturers and software versions, comparing data from different institutions can be challenging.

Additionally, domain adaptation/generalization techniques could be explored to enhance further the generalizability and robustness of radiomic features across diverse imaging conditions. Given the strong results achieved with feature-based methods like ComBat, statistical feature-based methods could be integrated with deep features [8] to limit the disparities caused by scanners and protocols.

## Conclusions

In summary, the variations in CT scanner settings significantly influence radiomic features, impacting their reliability for clinical tasks. Our study shows the effectiveness of harmonization techniques like ComBat and GANs to mitigate these variations, enhancing the reproducibility stability, and discriminative power of radiomic features in personalized medicine. By integrating these methodologies, we aim to refine the robustness of radiomics analysis, ensuring that these biomarkers remain consistent and discriminative across different scanners and protocol settings.

## Supporting information

S1 File S1 Fig. UMAP plots with each subplot showing 360 radiomic features samples (from 30 scans, 4 ROIs and original/harmonized/reference features), described by 93 radiomic features, and across harmonization methods for Group 1. S2 Fig. UMAP plots with each subplot showing 360 radiomic features samples (from 30 scans, 4 ROIs and original/harmonized/reference features), described by 93 radiomic features, and across harmonization methods for Group 2. S3 Fig. UMAP plots with each subplot showing 360 radiomic features samples (from 30 scans, 4 ROIs and original/harmonized/reference features), described by 93 radiomic features, and across harmonization methods for Group 3. S4 Fig. UMAP plots with each subplot showing 360 radiomic features samples (from 30 scans, 4 ROIs and original/harmonized/reference features), described by 93 radiomic features, and across harmonization methods for Group 4. S5 Fig. UMAP plots with each subplot showing 360 radiomic features samples (from 30 scans, 4 ROIs and original/harmonized/reference features), described by 93 radiomic features, and across harmonization methods for Group 5. S6 Fig. UMAP plots with each subplot showing 360 radiomic features samples (from 30 scans, 4 ROIs and original/harmonized/reference features), described by 93 radiomic features, and across harmonization methods for Group 6. S7 Fig. UMAP plots with each subplot showing 360 radiomic features samples (from 30 scans, 4 ROIs and original/harmonized/reference features), described by 93 radiomic features, and across harmonization methods for Group 8. S8 Fig. Samples of generated images from GAN harmonization. S9 Fig. Probability Density Function (PDF) plots showing the distribution of selected radiomic features across harmonization methods (ComBat, GAN, GAN+ComBat) for different ROIs. Features displayed per ROI include: Each plot compares the original (O, blue), harmonized (H, red), and reference (R, green) feature distributions, highlighting the effect of each method on radiomic features. S1 Table. Group-Wise and ROI-Specific Classification Scores: AUC scores for Non-Harmonized Radiomic Features S2 Table. Group-Wise and ROI-Specific Classification Scores: AUC scores for ComBat harmonization S3 Table. Group-Wise and ROI-Specific Classification Scores: AUC scores for GAN harmonization S4 Table. Group-Wise and ROI-Specific Classification Scores: AUC scores for GAN+ComBat harmonization S5 Table. Pairwise Wilcoxon signed-rank test Bonferroni corrected p-values comparing AUC differences among harmonization methods
(DOCX)
