# Supplementary material for: Harmonizing CT scanner acquisition variability in an anthropomorphic phantom: A comparative study of image-level and feature-level harmonization using GAN, ComBat, and their combination
Source: PLoS One. 2025 May 9;20(5):e0322365. doi: 10.1371/journal.pone.0322365 (PMC12063804; doi:10.1371/journal.pone.0322365)
Supplement: S1 File — S1 Fig. UMAP plots with each subplot showing 360 radiomic features samples (from 30 scans, 4 ROIs and original/harmonized/reference features), described by 93 radiomic features, and across harmonization methods for Group 1. S2 Fig. UMAP plots with each subplot showing 360 radiomic features samples (from 30 scans, 4 ROIs and original/harmonized/reference features), described by 93 radiomic features, and across harmonization methods for Group 2. S3 Fig. UMAP plots with each subplot showing 360 radiomic features samples (from 30 scans, 4 ROIs and original/harmonized/reference features), described by 93 radiomic features, and across harmonization methods for Group 3. S4 Fig. UMAP plots with each subplot showing 360 radiomic features samples (from 30 scans, 4 ROIs and original/harmonized/reference features), described by 93 radiomic features, and across harmonization methods for Group 4. S5 Fig. UMAP plots with each subplot showing 360 radiomic features samples (from 30 scans, 4 ROIs and original/harmonized/reference features), described by 93 radiomic features, and across harmonization methods for Group 5. S6 Fig. UMAP plots with each subplot showing 360 radiomic features samples (from 30 scans, 4 ROIs and original/harmonized/reference features), described by 93 radiomic features, and across harmonization methods for Group 6. S7 Fig. UMAP plots with each subplot showing 360 radiomic features samples (from 30 scans, 4 ROIs and original/harmonized/reference features), described by 93 radiomic features, and across harmonization methods for Group 8. S8 Fig. Samples of generated images from GAN harmonization. S9 Fig. Probability Density Function (PDF) plots showing the distribution of selected radiomic features across harmonization methods (ComBat, GAN, GAN+ComBat) for different ROIs. Features displayed per ROI include: Each plot compares the original (O, blue), harmonized (H, red), and reference (R, green) feature distributions, highlighting the effect of each method on rad [file pone.0322365.s001.docx]

# Supplementary material:


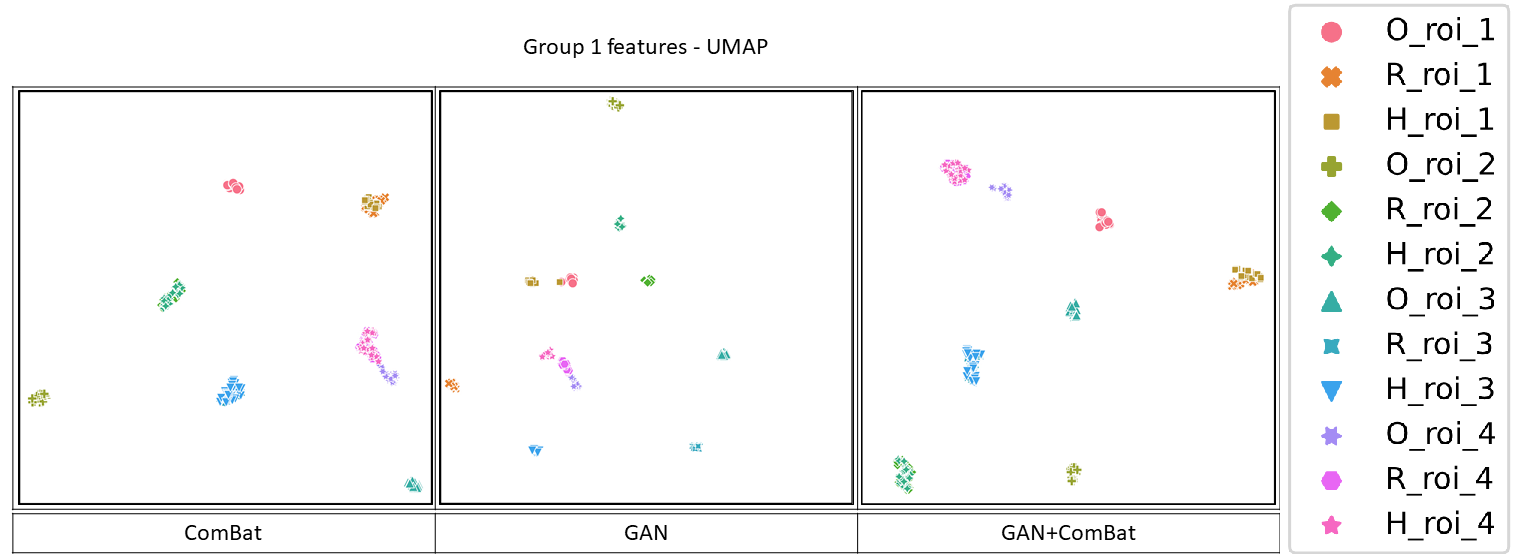


**S1 Fig. UMAP plots with each subplot showing 360 radiomic features samples (from 30 scans, 4 ROIs and original/harmonized/reference features), described by 93 radiomic features, and across harmonization methods for Group 1.**


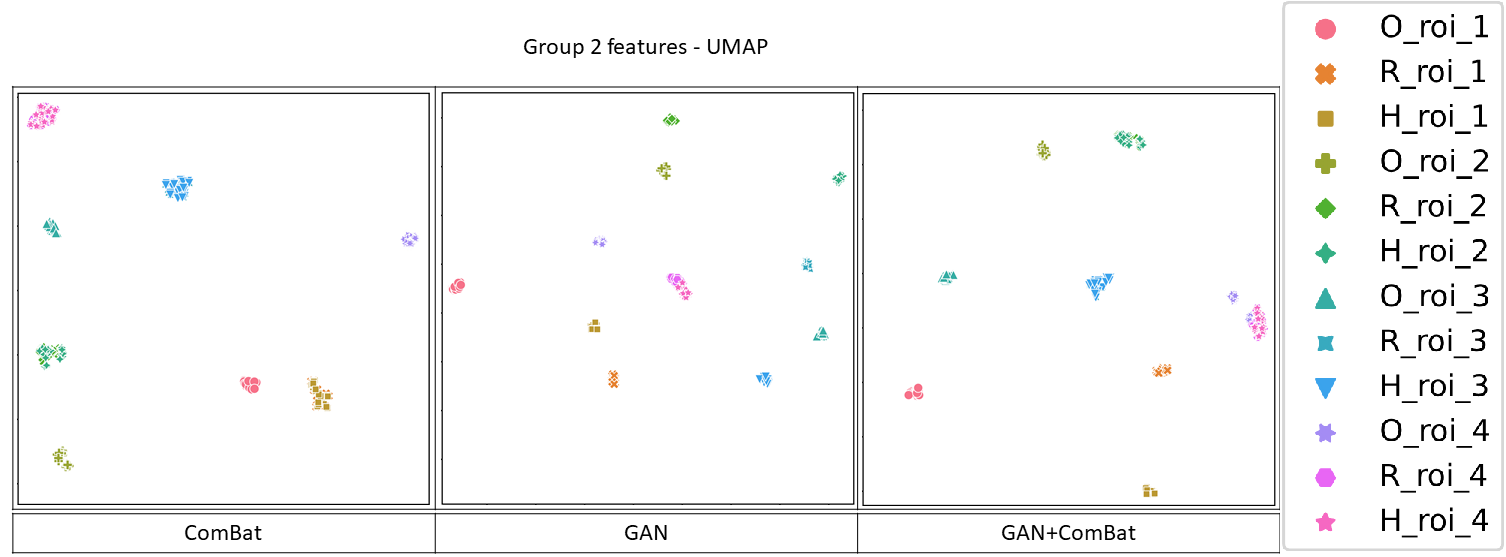


**S2 Fig. UMAP plots with each subplot showing 360 radiomic features samples (from 30 scans, 4 ROIs and original/harmonized/reference features), described by 93 radiomic features, and across harmonization methods for Group 2.**


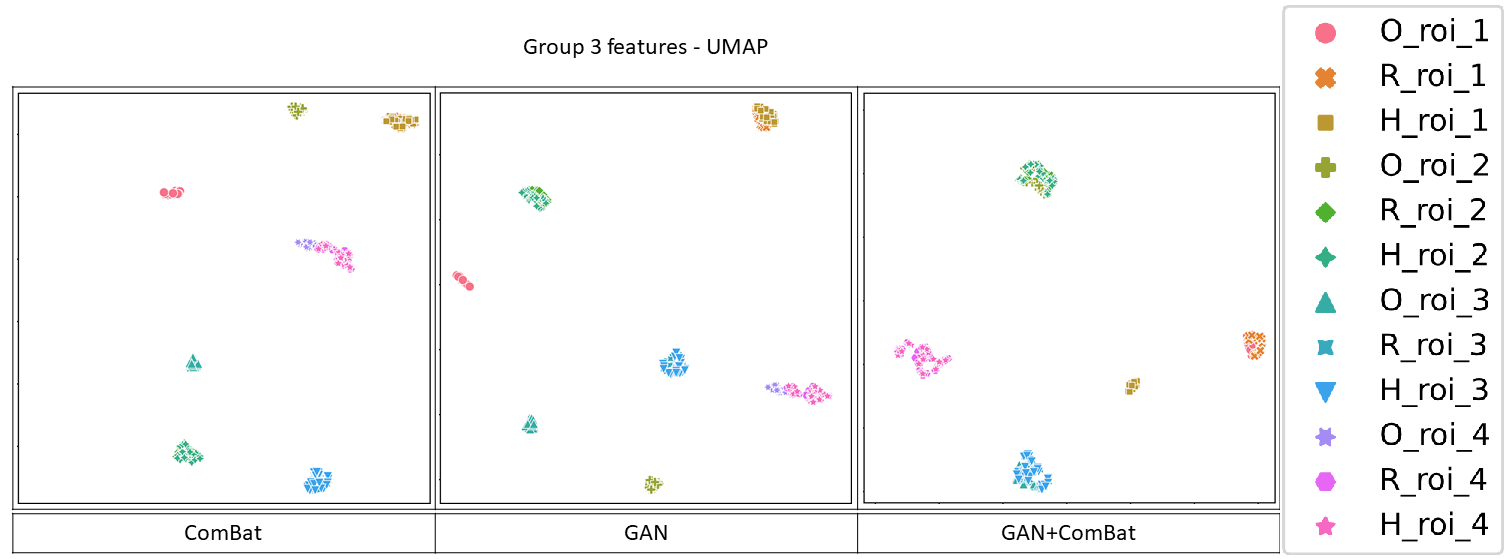


**S3 Fig. UMAP plots with each subplot showing 360 radiomic features samples (from 30 scans, 4 ROIs and original/harmonized/reference features), described by 93 radiomic features, and across harmonization methods for Group 3.**


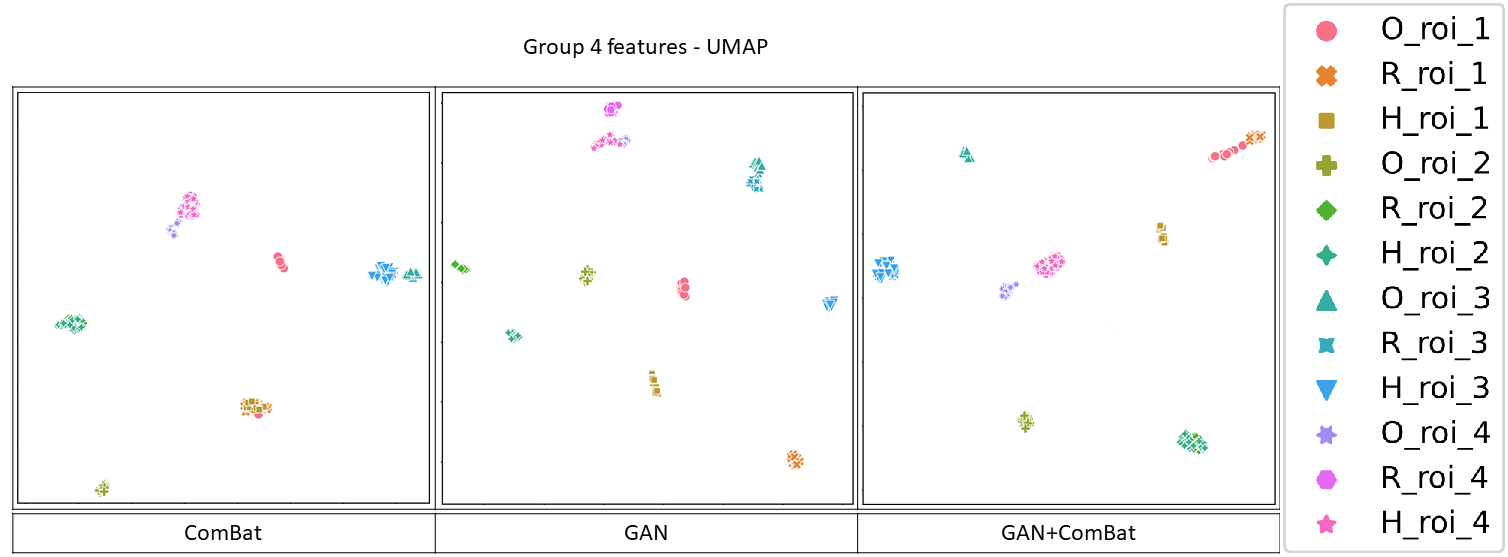


**S4 Fig. UMAP plots with each subplot showing 360 radiomic features samples (from 30 scans, 4 ROIs and original/harmonized/reference features), described by 93 radiomic features, and across harmonization methods for Group 4.**


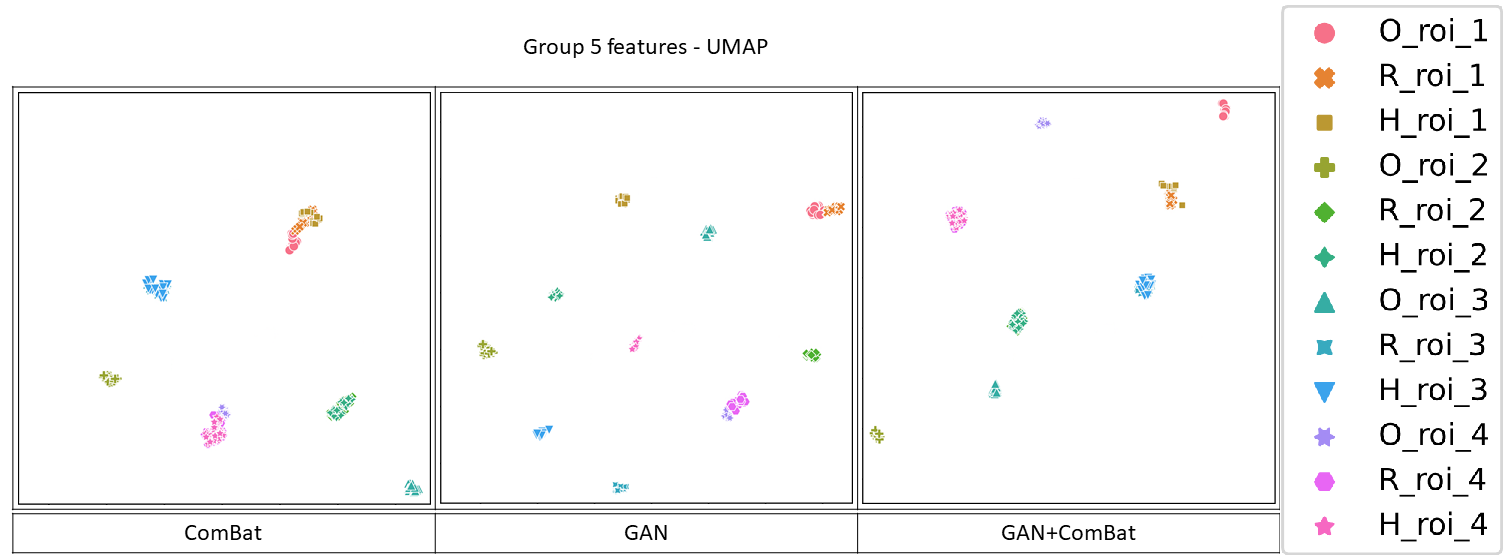


**S5 Fig. UMAP plots with each subplot showing 360 radiomic features samples (from 30 scans, 4 ROIs and original/harmonized/reference features), described by 93 radiomic features, and across harmonization methods for Group 5.**


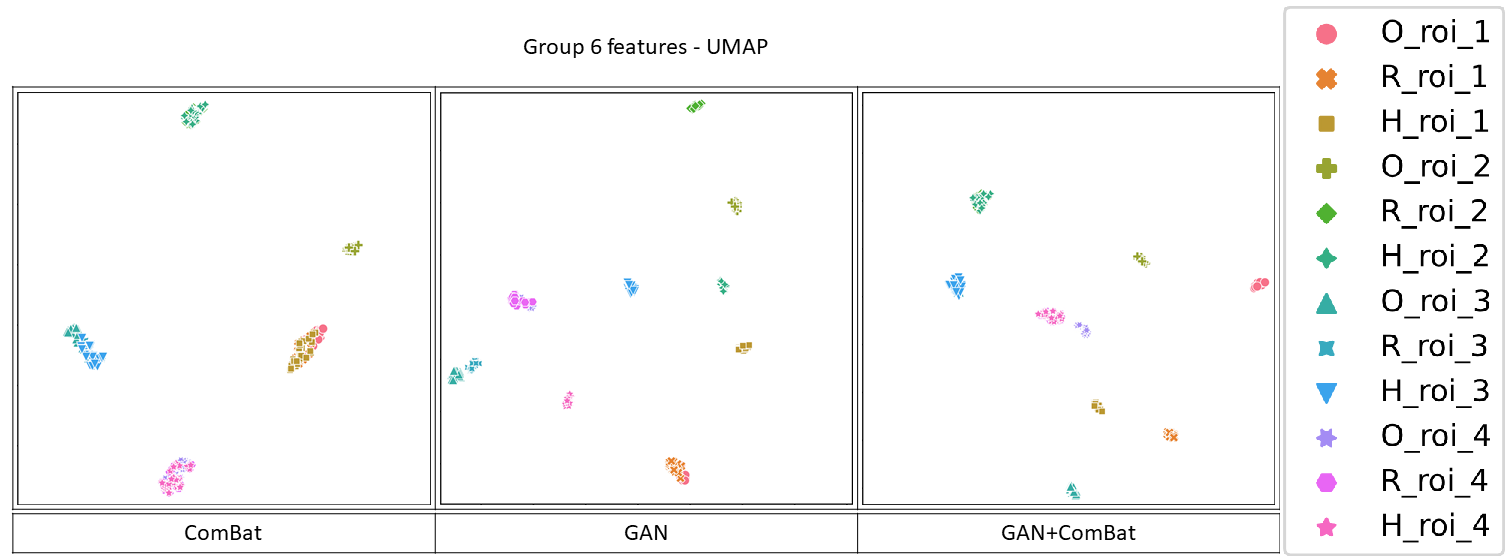


**S6 Fig. UMAP plots with each subplot showing 360 radiomic features samples (from 30 scans, 4 ROIs and original/harmonized/reference features), described by 93 radiomic features, and across harmonization methods for Group 6.**


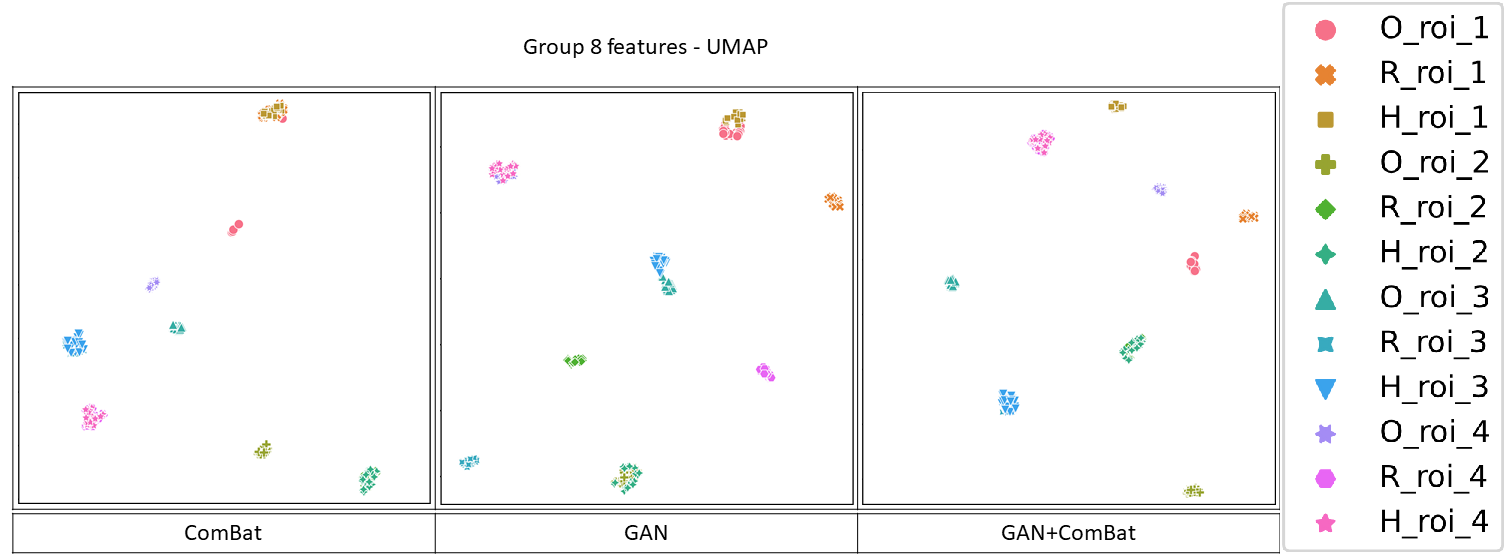


**S7 Fig. UMAP plots with each subplot showing 360 radiomic features samples (from 30 scans, 4 ROIs and original/harmonized/reference features), described by 93 radiomic features, and across harmonization methods for Group 8.**

**S1 Table. Group-Wise and ROI-Specific Classification Scores: AUC scores for Non-Harmonized Radiomic Features**

| **Groups** | **ROI_1** | **ROI_2** | **ROI_3** | **ROI_4** | **Average group AUC** |
| --- | --- | --- | --- | --- | --- |
| **Group 1** | 0.68 | 0.36 | 0.36 | 0.16 | 0.38 |
| **Group 2** | 1.00 | 0.91 | 0.92 | 0.96 | 0.94 |
| **Group 3** | 0.95 | 0.87 | 0.86 | 0.95 | 0.90 |
| **Group 4** | 0.89 | 0.79 | 0.79 | 0.89 | 0.84 |
| **Group 5** | 0.68 | 0.36 | 0.36 | 0.84 | 0.56 |
| **Group 6** | 1.00 | 0.86 | 0.86 | 1.00 | 0.93 |
| **Group 7** | 0.95 | 0.86 | 0.86 | 1.00 | 0.92 |
| **Group 8** | 0.85 | 0.79 | 0.79 | 0.94 | 0.84 |
| **Average ROI AUC** | 0.88 | 0.73 | 0.73 | 0.84 | 0.79 |

**S2 Table. Group-Wise and ROI-Specific Classification Scores: AUC scores for ComBat harmonization**

| **Groups** | **ROI_1** | **ROI_2** | **ROI_3** | **ROI_4** | **Average group AUC** |
| --- | --- | --- | --- | --- | --- |
| **Group 1** | 1.00 | 0.81 | 0.81 | 0.94 | 0.88 |
| **Group 2** | 1.00 | 0.91 | 0.92 | 0.96 | 0.94 |
| **Group 3** | 0.95 | 0.87 | 0.86 | 0.95 | 0.90 |
| **Group 4** | 1.00 | 0.86 | 0.87 | 0.92 | 0.91 |
| **Group 5** | 1.00 | 0.78 | 0.78 | 0.89 | 0.86 |
| **Group 6** | 1.00 | 0.86 | 0.86 | 1.00 | 0.93 |
| **Group 7** | 0.95 | 0.86 | 0.86 | 1.00 | 0.92 |
| **Group 8** | 1.00 | 0.86 | 0.86 | 0.95 | 0.92 |
| **Average ROI AUC** | 0.98 | 0.85 | 0.85 | 0.95 | 0.91 |

**S3 Table. Group-Wise and ROI-Specific Classification Scores: AUC scores for GAN harmonization**

| **Groups** | **ROI_1** | **ROI_2** | **ROI_3** | **ROI_4** | **Average group AUC** |
| --- | --- | --- | --- | --- | --- |
| **Group 1** | 0.39 | 0.39 | 0.39 | 0.83 | 0.5 |
| **Group 2** | 1.00 | 0.93 | 0.94 | 0.97 | 0.96 |
| **Group 3** | 0.95 | 0.87 | 0.86 | 0.95 | 0.91 |
| **Group 4** | 0.83 | 0.75 | 0.80 | 0.88 | 0.81 |
| **Group 5** | 0.36 | 0.36 | 0.36 | 0.92 | 0.3 |
| **Group 6** | 1.00 | 0.91 | 0.91 | 1.00 | 0.95 |
| **Group 7** | 0.95 | 0.83 | 0.84 | 1.00 | 0.92 |
| **Group 8** | 0.85 | 0.77 | 0.77 | 0.94 | 0.82 |
| **Average ROI AUC** | 0.79 | 0.73 | 0.73 | 0.832 | 0.77 |

**S4 Table. Group-Wise and ROI-Specific Classification Scores: AUC scores for GAN+ComBat harmonization**

| **Groups** | **ROI_1** | **ROI_2** | **ROI_3** | **ROI_4** | **Average group AUC** |
| --- | --- | --- | --- | --- | --- |
| **Group 1** | 1.00 | 0.86 | 0.86 | 0.95 | 0.92 |
| **Group 2** | 1.00 | 0.93 | 0.94 | 0.97 | 0.96 |
| **Group 3** | 0.95 | 0.87 | 0.86 | 0.95 | 0.91 |
| **Group 4** | 1.00 | 0.86 | 0.89 | 0.92 | 0.92 |
| **Group 5** | 1.00 | 0.83 | 0.84 | 0.95 | 0.91 |
| **Group 6** | 0.96 | 0.88 | 0.89 | 1.00 | 0.93 |
| **Group 7** | 0.95 | 0.83 | 0.84 | 1.00 | 0.92 |
| **Group 8** | 1.00 | 0.88 | 0.89 | 0.96 | 0.93 |
| **Average ROI AUC** | 0.98 | 0.87 | 0.88 | 0.96 | 0.92 |

**S5 Table. Pairwise Wilcoxon signed-rank test Bonferroni corrected p-values comparing AUC differences among harmonization methods**

| **Comparison** | **p-value** |
| --- | --- |
| **Non-harmonized vs ComBat** | 0.0026 |
| **Non-harmonized vs GAN** | 1.0000 |
| **Non-harmonized vs GAN+ComBat** | 0.0016 |
| **ComBat vs GAN** | 0.0098 |
| **ComBat vs GAN+ComBat** | 0.1031 |
| **GAN vs GAN+ComBat** | 0.0043 |


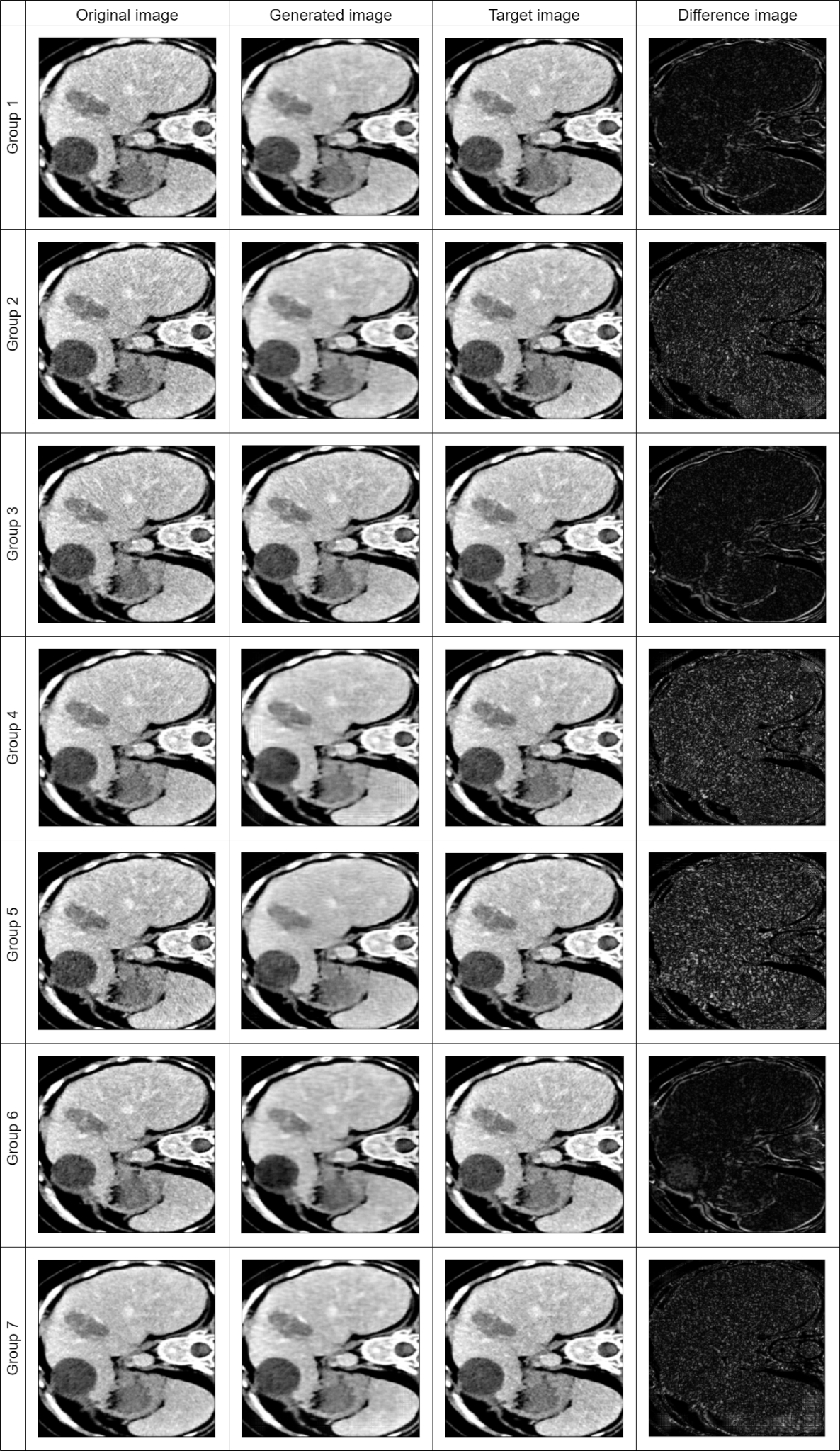


**S8 Fig. Samples of generated images from GAN harmonization**

**
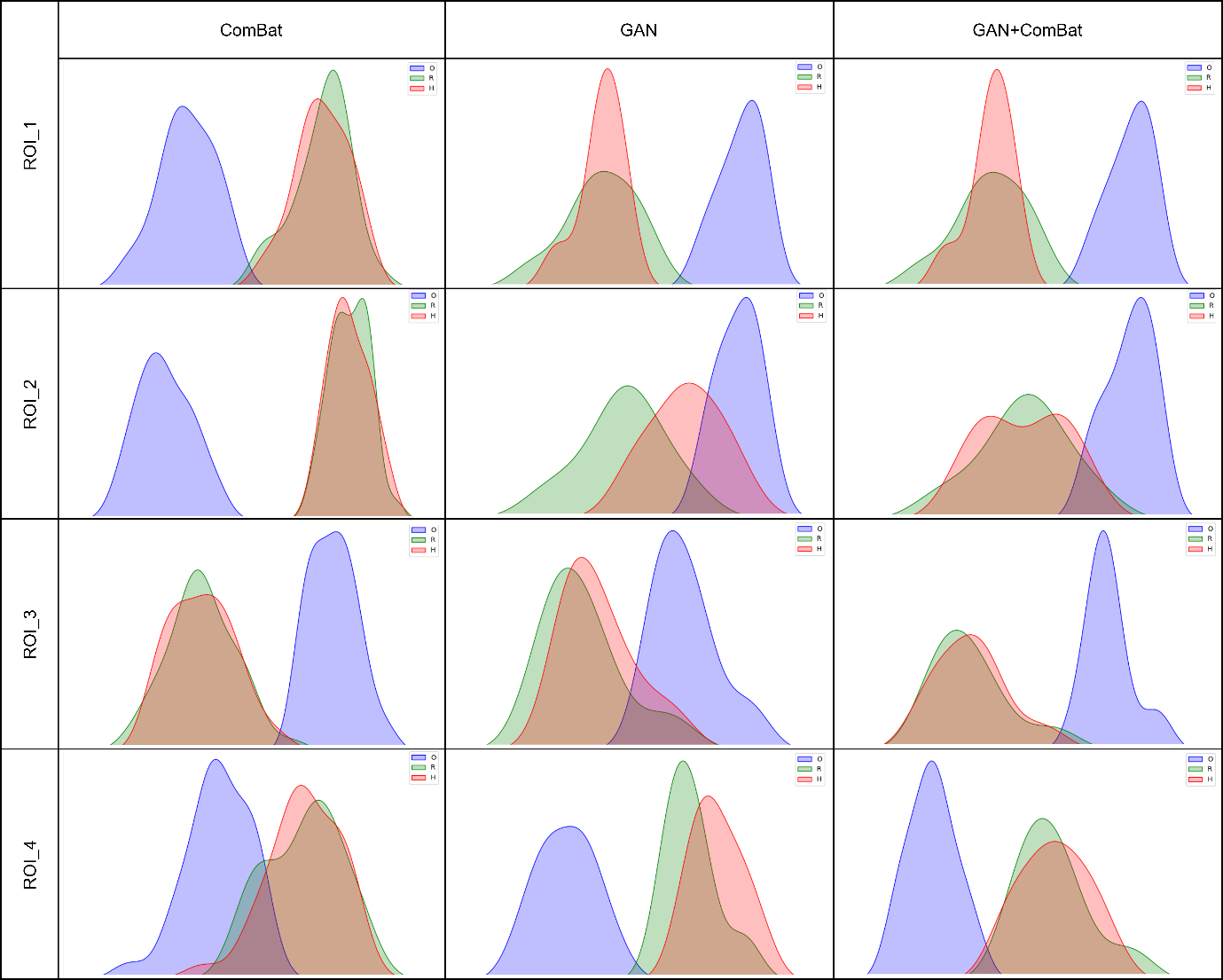
**

**S9 Fig. Probability Density Function (PDF) plots showing the distribution of selected radiomic features across harmonization methods (ComBat, GAN, GAN+ComBat) for different ROIs. Features displayed per ROI include:**

- **ROI 1: First order 90^th^ Percentile (Groups, 8, 5, 3 respectively in the first row)**
- **ROI 2: GLRLM Run Entropy (Groups 4, 3, 6 respectively in the second row)**
- **ROI 3: GLDM Gray Level Variance (Groups, 1, 3, 2 respectively in the third row)**
- **ROI 4: GLCM Correlation (Groups 5, 1, 2 respectively in the fourth row)**

**Each plot compares the original (O, blue), harmonized (H, red), and reference (R, green) feature distributions, highlighting the effect of each method on radiomic features.**
